# Properties and Biocompatibility of Chitosan and Silk Fibroin Blend Films for Application in Skin Tissue Engineering

**DOI:** 10.1100/2012/697201

**Published:** 2012-05-22

**Authors:** Witoo Luangbudnark, Jarupa Viyoch, Wiroon Laupattarakasem, Palakorn Surakunprapha, Pisamai Laupattarakasem

**Affiliations:** ^1^Department of Pharmacology, Faculty of Medicine, Khon Kaen University, Khon Kaen 40002, Thailand; ^2^Department of Pharmaceutical Technology, Faculty of Pharmaceutical Sciences, Naresuan University, Phitsanulok 65000, Thailand; ^3^Department of Orthopaedics, Faculty of Medicine, Khon Kaen University, Khon Kaen 40002, Thailand; ^4^Department of Surgery, Faculty of Medicine, Khon Kaen University, Khon Kaen 40002, Thailand

## Abstract

Chitosan/silk fibroin (CS/SF) blend films were prepared and evaluated for feasibility of using the films as biomaterial for skin tissue engineering application. Fourier transform infrared spectroscopy and differential scanning calorimetry analysis indicated chemical interaction between chitosan and fibroin. Chitosan enhanced **β**-sheet conformation of fibroin and resulted in shifting of thermal degradation of the films. Flexibility, swelling index, and enzyme degradation were also increased by the chitosan content of the blend films. Biocompatibility of the blend films was determined by cultivation with fibroblast cells. All films showed no cytotoxicity by XTT assay. Fibroblast cells spread on CS/SF films via dendritic extensions, and cell-cell interactions were noted. Cell proliferation on CS/SF films was also demonstrated, and their phenotype was examined by the expression of collagen type I gene. These results showed possibility of using the CS/SF films as a supporting material for further study on skin tissue engineering.

## 1. Introduction

Although autograft is the gold standard for the treatment of full-thickness wounds, its application is limited by constraint of available donor skin. On the other hand, graft versus host reaction and risks of disease transfer are concerned with in using allograft [1–3]. Due to these limitations, skin substitute is developed under the tissue engineering principle, which consolidates knowledge of engineering and life sciences to develop biological materials that can replace, maintain, or improve tissue functions [1, 4]. In order to construct biological substitutes holding potential to repair or replace damaged tissues, appropriate* cell*s and *scaffold* are required [5].

Skin substitute is primarily designed as acellular material acting as barrier and stimulating neoskin formation [2, 6]. Unfortunately, it cannot provide the same biochemical stimulus as skin and, therefore, cellular material is developed. The cellular skin substitute can be constructed by culturing skin cells together with the scaffold [5, 7].

The cultured cells have limited capacity to reform their specific architecture. It is the scaffold that acts as the artificial extracellular matrix (ECM) to provide the template for supporting cell attachment, guides cell proliferation and differentiation, and also serves as a carrier for the transportation of cells into the defect site [8–10]. Currently, a number of natural and synthetic polymers are being used as the scaffold. The natural polymers are preferred because the synthetic origin lacks cell-recognition signals [11]. Therefore, silk fibroin (SF) and chitosan (CS), which are natural materials, are selected for fabrication as the scaffold in this study.

SF, a core protein element of silk fiber, has been used as a biomaterial for medical applications because of its mechanical properties, biocompatibility and biodegradability [12, 13]. SF scaffold can support several cell types such as osteoblast-like cells, bone marrow stromal cells [12], keratinocytes, and dermal fibroblast cells [14, 15]. However, this biomaterial is very brittle in dry state and is difficult to handle. Therefore, another polymer such as CS is added to the SF formulation. CS has been investigated for its biocompatibility, biodegradability, and toxicity in its use as a scaffold in tissue engineering [16, 17]. As a skin substitute, CS-based scaffolds can support keratinocyte and fibroblast attachment and their proliferation [18–20]. Nonetheless, pure CS scaffold rapidly degrades and has a high swelling property in aqueous solution.

In order to avoid the exclusive limitations of pure SF and CS, blending of both materials was suggested with proven miscibility between CS and SF blend films [21–24]. However, some specific properties and the biocompatibility of the CS/SF blend films as a scaffold in skin tissue engineering are still unexplored. In this study, the CS/SF blend films were prepared by blending SF and CS with high degree of deacetylation (DD). Beside the improvement of mechanical properties, CS with high DD was reported to have good cytocompatibility and low inflammatory reactions [25]. The physicochemical characteristics of the blend films were investigated by measuring various properties such as tensile strength, swelling index, and enzymatic degradation. Biocompatibility was further determined by cultivation with fibroblast cells using cell adhesion and proliferation as indicators. Furthermore, the fibroblast phenotype was confirmed by the expression of collagen type I gene.

## 2. Materials and Methods

### 2.1. Materials

Cocoons of *Bombyx mori* Thai silkworm (Nangnoi srisaket1) were obtained from Queen Sirikit Sericulture Center, Chaiyaphum Province, Thailand. CS derived from shrimp shell (MW 100,000, DD ≥ 95%) was purchased from Seafresh, Bangkok, Thailand. Lysozyme from hen egg white was purchased from Fluka Chemie GmbH, Buchs, Switzerland. Dulbecco's modified eagle medium (DMEM) and other chemicals for cell culture were purchased from Sigma-Aldrich, Missouri, USA. Cell proliferation kit II (sodium 3′-[1-(phenylaminocarbonyl)-3,4-tetrazolium]-bis (4-methoxy-6-nitro) benzene sulfonic acid hydrate, XTT) was purchased from Roche Diagnostics GmbH, Manheim, Germany.

### 2.2. Preparation of Regenerated Silk Fibroin from *Bombyx mori Cocoons*

SF was isolated from *B. mori* silkworm cocoons as previously described with some modification [26]. Briefly, small pieces of silk cocoon were boiled in water at 90°C for 150 min and in 25 mM sodium hydroxide at 70°C for 30 min, respectively. After air drying, the resultant fibrous tissue was solubilized in 3.0 M calcium chloride solution at 90°C. Calcium chloride residue was removed by dialysis, using cellulose tube against distilled water at 4°C for 72 h, and then centrifuged at 8000 ×g for 15 min to remove foreign particles. The retentive solution was lyophilized to obtain the regenerated SF and kept in desiccators until used.

### 2.3. Preparation of the CS/SF Blend Films

The CS/SF blend films were prepared by casting the mixture of 2 wt% of each SF and CS solution, which were dissolved in lactic acid solution (pH 4.0). The CS solution was blended with the SF solution in various ratios (CF) at 3 : 1, 2 : 1, 1 : 1, 1 : 2 and 1 : 3. Subsequently, the blended solution was casted into a mold and allowed to dry at 37–40°C. The resulting films were immersed in 0.21 M NH_4_OH in methanol and then repeatedly rinsed with phosphate buffer saline (PBS) until the neutral pH was obtained and was further dried at 37–40°C. The dried films were stored in desiccators until used.

### 2.4. Physicochemical Properties of CS/SF Blend Films

#### 2.4.1. Scanning Electron Microscope (SEM)

The surface morphology of the CS/SF films was observed using SEM (Model 1455VP, LEO Electron Microscopy, Cambridge, England). All the test films were coated with an ultrathin gold layer, and the morphology was observed at a magnification of 3500x.

#### 2.4.2. Fourier Transform Infrared (FTIR) Spectroscopy

The infrared spectra of the blend films were used to investigate chemical conformation and miscibility of CS and SF in the blends at spectral region of 600–2000 cm^−1^ by using Model GX series, Perkin Elmer, USA at room temperature.

#### 2.4.3. Differential Scanning Calorimetry (DSC)

The thermal behavior of the blend films was performed with a thermal analysis instrument (DSC1 STAR System, Mettler Toledo, Switzerland) at a heating rate of 10°C/min and nitrogen gas flow rate at 50 mL/min.

#### 2.4.4. Mechanical Properties

Mechanical properties were used to determine the performance of materials expected to undergo stresses during utilization. The tensile strength and percent elongation at the breakage of the blend films were determined at dry state. Samples were cut into rectangular (10 mm × 30 mm) pieces, and average thickness of four different locations was measured with a micrometer. The tensile properties of the blend films were examined with a tensometer (Instron 8872, Instron Ltd., UK) at constant rate (15 mm/min).

#### 2.4.5. Swelling Property

Water absorption property of the CS/SF blend films was determined by immersion in PBS (pH 7.4) at 37°C for 24 h. The wet weight (*W*_*s*_, swollen weight) and dry weight (*W*_*d*_, dried at 65°C overnight) were measured. Then, the swelling index was calculated as shown in (1).

(1) $$\text{Swelling}\;\text{index}\;=\frac{\left(W_{s}-W_{d}\right)\;\times 100}{W_{d}}.$$

#### 2.4.6. In Vitro Enzymatic Degradation

Degradation of the CS/SF blend films was determined as percentage of weight remained after incubation in lysozyme solution. According to Nwe et al. [27], samples with known dry weight (*W*_*o*_) were immersed in PBS pH 7.4 containing 10 *μ*g/mL lysozyme at 37°C for 4 weeks and refreshed weekly. At various time points (1, 2, 3, and 4 weeks), the samples were removed and dried at 65°C overnight. The dried samples were weighed and determined as dry weight after degradation (*W*_*t*_) and the percentage of the remaining weight was calculated as shown in (2).

(2) $$\text{\%}\;\text{Weight}\;\text{remained}\;=\;\left(\frac{W_{t}}{W_{o}}\right)\;\times \;100.$$

### 2.5. Biocompatibility of Fibroblast Cells and CS/SF Blend Films

#### 2.5.1. Indirect Cytotoxicity Test

Before culture, the CS/SF blend films were sterilized with 75% ethanol and immersed in fresh culture medium for 24 h. Biocompatibility was tested with human dermal fibroblasts (HDF; PromoCell, Germany), for which the 7–11 passages of HDF cells were used.

The cytotoxicity test of the CS/SF blend films was adapted from the ISO10993-5 [28], an international standard method for testing medical devices. The cell viability was determined after being incubated with the extraction medium from the prepared film. The extraction medium was obtained by incubating the sterile CS/SF blend films at 37°C in fresh culture medium at extraction ratio of 10 mg/mL. After 24 h, the extraction medium was diluted to final concentration of 0.5, 1, and 2.5 mg/mL. The fibroblast cells were seeded in 96-well plate at a density of 1×10^4^ cells/well and incubated in culture medium for 24 h, then replaced with various concentrations of extraction medium, and cultured for further 5 days without any change of medium. The XTT assay was used to determine cell viability based on the cleavage of the yellow tetrasodium salt (XTT) by viable cells to form soluble orange formazan product that is directly proportional to the number of living cells and quantified by measuring its absorbance at 490 nm. The viability of cells cultured with normal culture medium in the same conditions was used as control and the absorption of the control was adjusted to 100%.

#### 2.5.2. Cell Adhesion and Proliferation Test

The sterile CS/SF blend films were placed into 96-well plastic plate. 1 × 10^4^ cells/well of fibroblast cells were seeded on the prepared film and then incubated at 37°C with 5% CO_2_. After 1, 7, and 14 days, the viability of cells was determined by using XTT assay.

#### 2.5.3. Cell Morphology

The morphology of fibroblast cells on the blend films was observed on day 3 by SEM. The samples were washed with PBS and subsequently fixed with 3.0% glutaraldehyde at 4°C for 4 h. Subsequently, they were dehydrated through a series of graded ethanol, air-dried overnight, and sputtered with gold for SEM observation.

#### 2.5.4. Collagen Type I Gene Expression

Total RNA was isolated from fibroblast cells cultured on the CS/SF films using the Trizol reagent. The one-step RT-PCR system was used for reverse transcription of RNA. Collagen type 1A1 and *β*-actin cDNA were amplified by C-1000 thermal cycler, Bio-Rad, CA, USA. The specific sense and antisense primers used for the reaction were designed from listed NIH GenBank database having the following sequences: type I collagen, sense: 5′ATGTTCAGCTTTGTGGACCTCCG-3′ and antisense: 5′-AACACCTTGCCGTTGTCGCA-3′; *β*-actin, sense: 5′TGGTGGGCATGGGTCAGAAGGATT-3′ and antisense: 5′-AGGGATAGCACAGCCTGGATAGCA-3′. PCR was conducted in a programmed temperature control system for 30 cycles under the following cycle condition: 15 s at 94°C for denaturation, 30 s at 60°C for annealing, and 1 min at 68°C for extension and 5 min at 72°C for final extension. The PCR products were run on 1% agarose gel.

### 2.6. Statistical Analysis

Data were presented as mean ± standard error (SE). Measurements were conducted in triplicate. A comparison between groups was analyzed with ANOVA, using SPSS 11.5 program. *P* < 0.05 was considered statistically significant.

## 3. Results

### 3.1. Film Appearance and Surface Morphology

**Figure 1:**
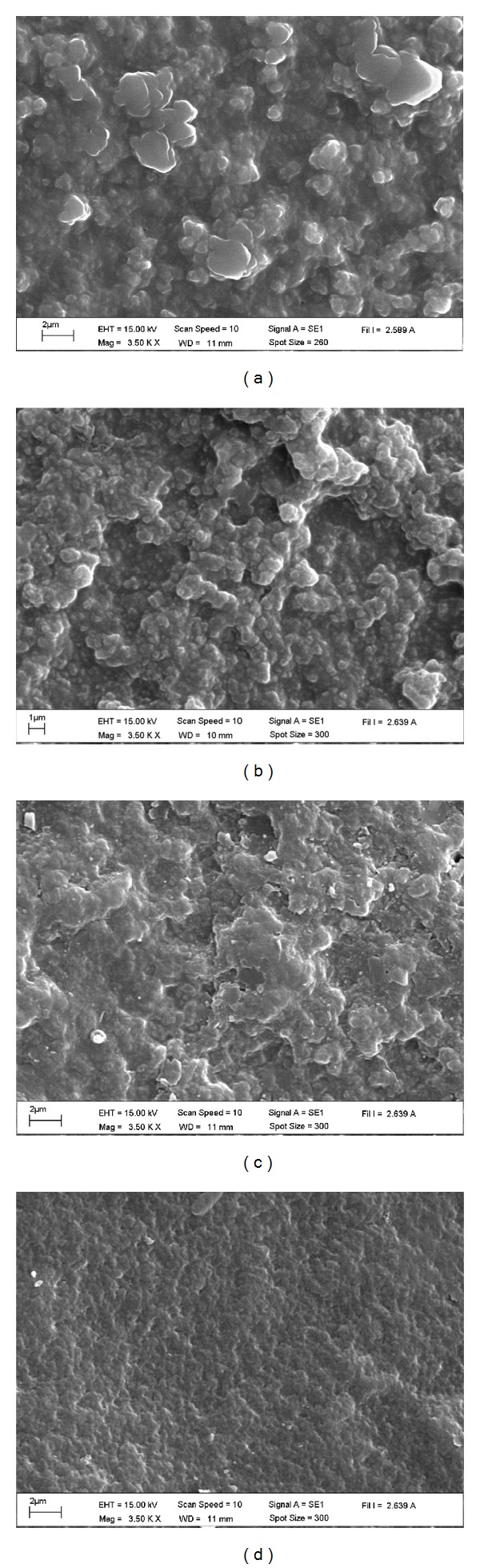
Surface morphology of CS/SF blend films with various blend ratios of chitosan : fibroin (CF) of (a) 3 : 1, (b) 2 : 1 (c) 1 : 1, and (d) 1 : 2 which was observed by SEM at 3500x magnification.

The appearance of the CS/SF blend films was uniform yellowish, 75 ± 25 *μ*m thick and strong enough to handle without deformation, except CF 1 : 3 formulation which was brittle and difficult to remove from the mold. Therefore, CF 1 : 3 films were not selected for further study. The surface morphology of the blend films was examined by SEM (Figure 1). SEM images of the dried blend films after immersion in PBS solution exhibited a nonporous surface which was dense and rough. Depending on CS : SF blend ratio, high CS content films CF 3 : 1 (Figure 1(a)) and CF 2 : 1 (Figure 1(b)) showed rougher surface with irregular bulges throughout the surfaces than the low CS content CF 1 : 1 (Figure 1(c)) and CF 1 : 2 (Figure 1(d)).

### 3.2. FTIR Characteristics

**Figure 2:**
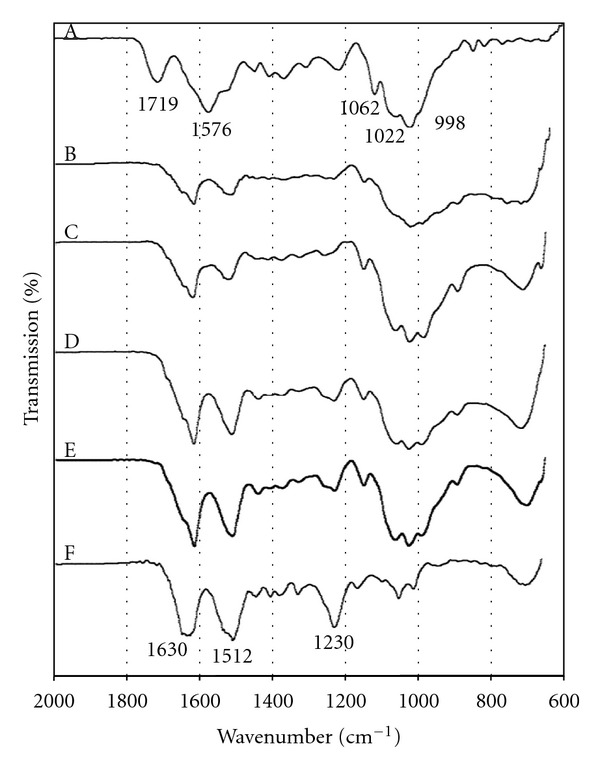
FTIR spectra of (A) CS film and CS/SF blend films with various blend ratios of chitosan : fibroin (CF) of (B) 3 : 1, (C) 2 : 1, (D) 1 : 1, (E) 1 : 2 and (F) SF film.

The FTIR spectra of the CS/SF blend films with different compositions of CS and SF were shown in Figure 2. CS film (Figure 2(A)) exhibited absorption band at 1719 cm^−1^ (C=O) and 1585 cm^−1^ (N-H), which were assigned to amino group, and absorption bands at 1123 and 865 cm^−1^ were attributed to the saccharide structure [29, 30]. SF film (Figure 2(F)) presented absorption bands at 1630 cm^−1^ (amide I) and 1512 cm^−1^ (amide II) which corresponds with the *β*-sheet conformation and another absorption band at 1233 cm^−1^ (amide III) assigned to random coil conformation [31]. This indicated that *β*-sheet and random coil are presented simultaneously in SF film.

The FTIR spectra of the blend samples showed in Figure 2(B–E) exhibited absorption bands of both SF and CS with intensity differences from varying composition of both materials. The blend films showed shifting of absorption bands of SF and disappearance of C=O and N–H groups of CS. The blend films displayed downward shifting of amide I absorption band of SF from 1630 to around 1620 cm^−1^ indicating that blending with CS further induced *β*-sheet structure; the new absorption shoulder of amide III at higher wave number around 1260 cm^−1^ presented conformation transition from random coil to *β*-sheet of SF [31, 32]. This structural change was markedly exhibited with the CF 1 : 2 blend film while the other blend films tended to present a less stable structure that was revealed as was previously reported [33, 34].

### 3.3. Thermal Behavior

**Figure 3:**
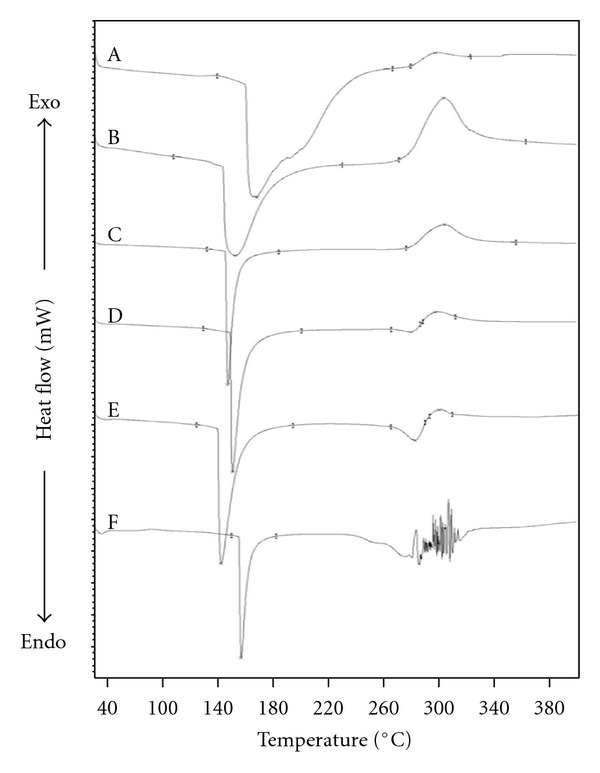
DSC thermograms of (A) CS film and CS/SF blend films with various blend ratios of chitosan : fibroin (CF) of (B) 3 : 1, (C) 2 : 1, (D) 1 : 1, (E) 1 : 2, and (F) SF film.

Thermal behavior of the blend films was investigated by DSC measurement as shown in Figure 3. The endothermic peak at temperature below 160°C in all samples attributed to the evaporation of moisture within the samples. The thermogram of CS film (Figure 3(A)) exhibited a characteristic exothermic peak at 297°C, which is ascribed to dehydration of saccharide rings, depolymerization, and decomposition of CS [34]. An endothermic peak at above 280°C of SF film (Figure 3(F)) attributed to the thermal degradation of SF film due to disintegration of intermolecular interaction. The DSC thermograms of the blend films (Figures 3(B)–(E)) showed mixed characteristic peaks of the two components, and thermal decomposition was not observed. In comparison to SF film, the blend films showed downward shifting of the moisture evaporation which reached maximum with the CF 1 : 2 blend film.

### 3.4. Mechanical Properties

**Figure 4:**
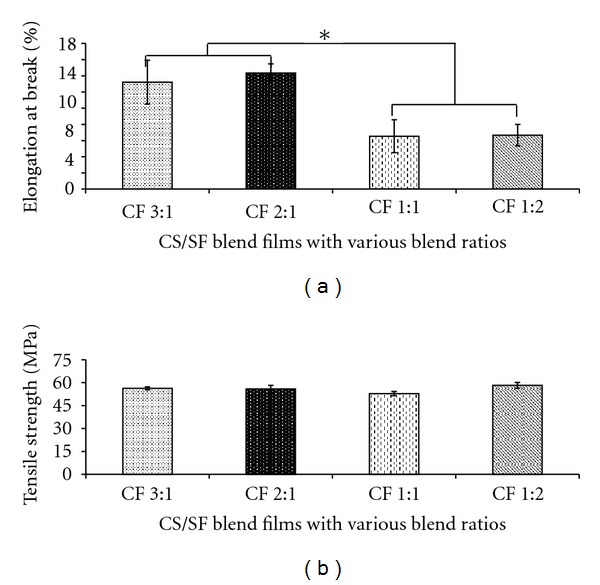
Mechanical properties of CS/SF blend films with various blend ratios of chitosan : fibroin (CF) at dry state; (a) % elongation at break and (b) tensile strength. Each bar represents mean ± standard error (*n* = 3). One-way ANOVA showed a significant difference in percent remaining weight among groups (*P < 0.05).

Mechanical properties of the blend films were investigated in terms of elongation at break and tensile strength at dry state as shown in Figure 4. The CS/SF blend films exhibited the percentage of elongation at break and tensile strength in range of 6.4% to 14.4% and 52.8 to 58.3 MPa, respectively. The elongation at break of the blend films (Figure 4(a)) was increased with increasing proportion of CS which indicated that the addition of CS was beneficial to enhancing the flexibility of the blend film. However, tensile strength of the blend films (Figure 4(b)) was not significantly different.

### 3.5. Swelling Property and Retain Ability

**Figure 5:**
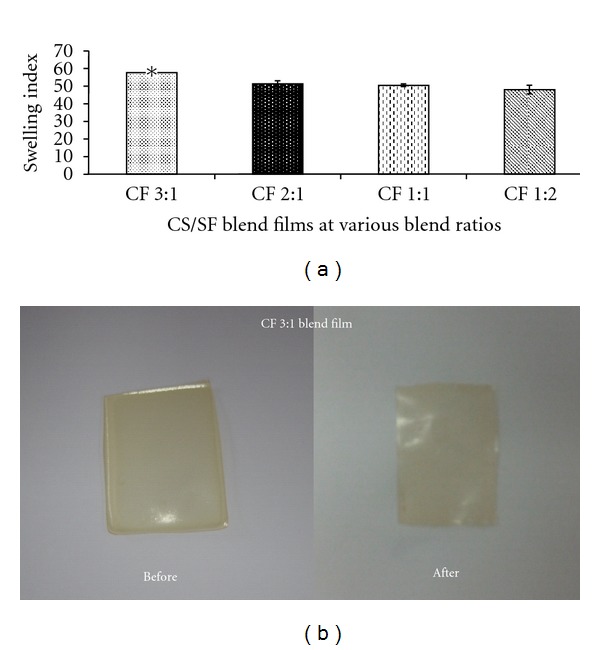
Swelling property and retain ability of CS/SF blend films with various blend ratios of chitosan : fibroin (CF) in PBS (pH 7.4) at 37°C for 24 h; (a) degree of swelling and (b) morphology. Each bar represents mean ± standard error (*n* = 3). One-way ANOVA showed a significant difference in degree of swelling among groups (*P < 0.05).

Water absorption ability and retain ability are other important factors in determining the usefulness of the biomaterials. The absorption ability of the CS/SF blend films was measured in terms of degree of swelling at equilibrium. It was found that the degree of swelling of the blend films was in range of 48–57% of their dry weight and relatively correlated with the CS content (Figure 5(a)). The prepared blend films could retain their form in aqueous solution. The CF 3 : 1 blend film which exhibited highest degree of swelling also retained its structure after immersion in PBS (pH 7.4) for 24 h as was shown in Figure 5(b).

### 3.6. In Vitro Enzymatic Degradation

**Figure 6:**
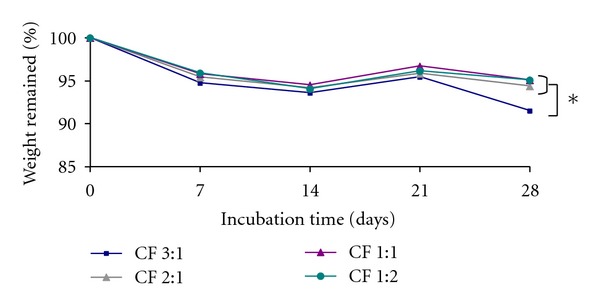
The percentages of remaining weight of CS/SF blend films with various blend ratios of chitosan : fibroin (CF) incubated in lysozyme solution at 37°C as a function of time. Each point represents mean ± standard error (*n* = 3). One-way ANOVA showed a significant difference in percent remaining weight among groups (*P < 0.05).

Degradation of the CS/SF blend films was mainly affected by the degradation of CS [35]. Therefore, the degradation behavior of the CS/SF blend films was studied *in vitro* by degradation with lysozyme, and the percentage of weight remained was determined (Figure 6). It was found that all samples could retain their structure over the study period and maintained more than 90% of their original weight after 4 weeks of incubation. It was noticed that the high-CS-content films (CF 3 : 1) showed a faster degradation rate.

### 3.7. Indirect Cytotoxicity Test

**Figure 7:**
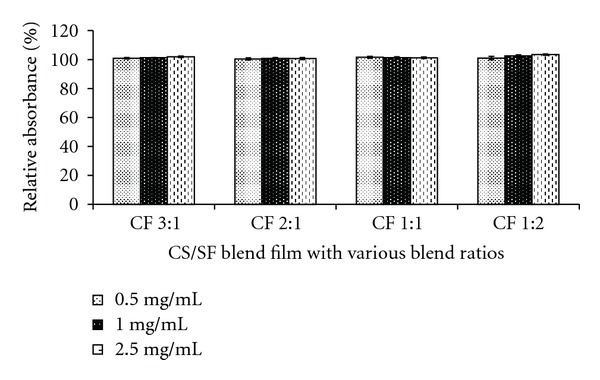
Viability of the fibroblast cells cultured with extraction media from different blend films. Data represent mean ± standard error (*n* = 3) (CF: chitosan :  fibroin ratio).

Cytotoxicity test has been accepted as the first criterion for biosafety assessment. To evaluate the potential of using the blend films in skin tissue engineering application, human dermal fibroblast cells (HDFs) were used as the reference cells. In this study, indirect cytotoxicity test was conducted by observing the viability of fibroblast cells cultured in different concentrations of the extraction medium from all CS/SF blend films. Cell viability was evaluated by using XTT, in which absorbance index is proportionally related to the number of living cells. It was found that the viability of fibroblast cells cultured in the extraction medium was not different from the cells cultured in fresh medium (Figure 7). The obtained results indicated that the extraction media from all samples did not contain any cytotoxic substances that produced adverse reaction to cells. Therefore, the CS/SF blend films could be used as scaffolds for further investigation.

### 3.8. Biocompatibility of CS/SF Blend Films

**Figure 8:**
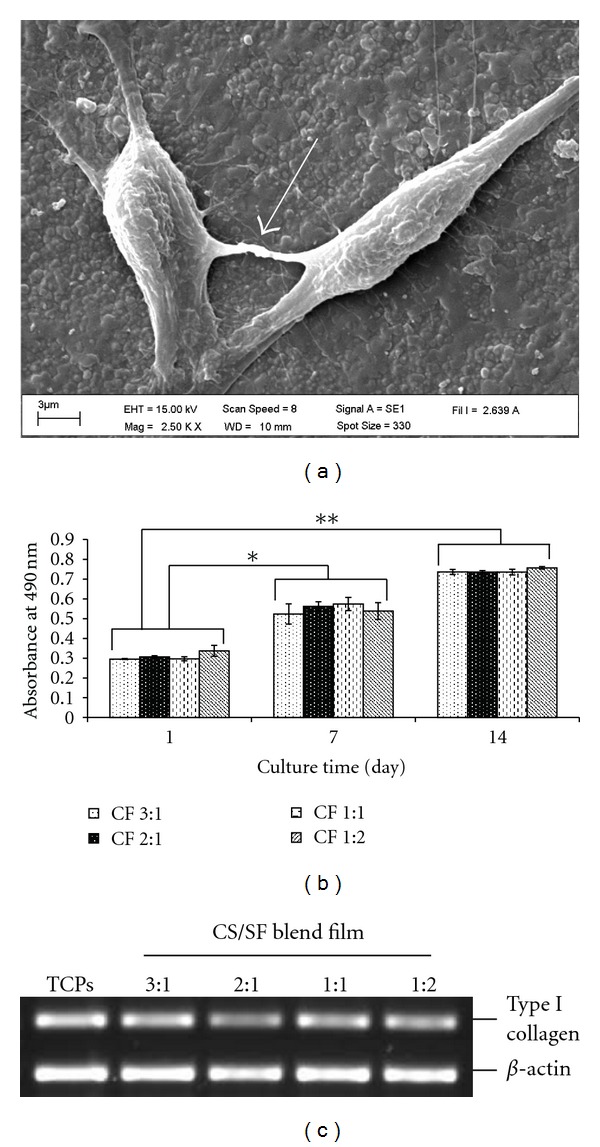
Biocompatibility of fibroblast cells on CS/SF blend films: (a) fibroblast cells adhesion on the blend film after 3 days of incubation, the white arrow showing cell-cell interaction, (b) fibroblast cells viability on the different blend films, and (c) type I collagen mRNA expression of fibroblast cells cultured on the blend films and tissue culture plate (TCPs) at day 14. Data represent mean ± standard error (*n* = 4). Student's *t*-test showed a significant difference in day 7 (*P < 0.05) and day 14 (**P < 0.001) as compared to day 1 (CF: chitosan : fibroin ratio).

The capability of the films to support fibroblast cell adhesion and proliferation represents the feasibility of their usage in skin tissue engineering. The fibroblast cells were cultured on the CS/SF blend films for 14 days, and the cell morphology, adhesion, viability, proliferation, and expression of collagen type I gene were determined (Figure 8).

Under the light microscope, the extension of cells could be observed within one day of incubation (data not shown). The morphology and adhesion of fibroblast cells on the CS/SF films were observed by SEM on day 3. The SEM images showed randomly distributed cells on the surface of the blend films. They exhibited typical spindlelike morphology and adhered tightly on the film surface by formation of lamellipodia and filopodia (Figure 8(a)), regarded as cell spreading, and the cell-cell connection was noted (arrow). The viability and proliferation of fibroblast cells on the blend films were evaluated using XTT assay. Up to 14 days of cultivation, the absorbance index of fibroblast cells cultured on the CS/SF blend films increased with the increasing culture period. There was no difference in the cell viability of all test groups (Figure 8(b)). This suggested that the CS/SF blend films could support fibroblast cells viability and proliferation.

In addition, the ability of the cultured fibroblast cells to maintain their function was observed. The expression of collagen type I gene, encoding for type I collagen, by fibroblast cells cultured on CS/SF blend films was analyzed and compared to the cells cultured on plastic plate (positive control), while the expression of *β*-actin was used as internal control. The result demonstrated that fibroblast cell cultures on all the CS/SF blend films could maintain their crucial function by expressing collagen type I gene as shown in Figure 8(c).

## 4. Discussion

Bioengineered skin substitutes were developed as an adjunctive or alternative use for skin grafts. By the tissue engineering principle, bioengineered skin can be prepared by culturing cells together with biomaterial scaffolds which are important in supporting cells in tissue construction. Hence, the scaffold should provide suitable physicochemical properties and biocompatibility to support cell proliferation.

 In this study, the CS/SF blend films were prepared and the morphology, physicochemical properties, and biocompatibility of the fibroblast cells on the blend films were investigated. The blend films showed nonporous rough surface. These globular structures become smaller as the SF content increased. This may be due to water absorption ability of CS that caused irregular bulging morphology after drying as was also shown by the swelling property of the blend. This rough surface was expected to support adhesion of fibroblast cells as was mentioned previously [36].

 FTIR spectroscopy was used to identify functional groups of CS and SF and their interaction between these two components in the blend films. SF film was simultaneously composed of both *β*-sheet and random coil structure. The *β*-sheet structure of SF film may be enhanced by the effect of acidic solvent during the preparation process [31]. In addition, transition from random coil to *β*-sheet of SF was increased by adding CS which was demonstrated by shifting of amide bands of SF. The conformation change of SF was supported by the interaction between CS and SF. As was found in the spectra of the blend films, the absorption band of the C=O and N-H group of CS disappeared and the amide bands of SF showed *β*-sheet structure by shifting amide I band of SF to lower wave number and a new absorption band at higher wave number of amide III was observed [31, 32]. This indicated intermolecular interaction between SF and CS. A previous report has shown that SF could form *β*-sheet structure by hydrogen bond between amide groups of SF and N-H of CS [37]. It is noticeable that the CF 1 : 2 blend films markedly exhibited *β*-sheet structure corresponding with the previous report [33, 34].

Intermolecular interaction between CS and SF was also demonstrated in thermal behavior of the blend films by increasing the decomposition temperature and decreasing moisture evaporation temperature. The reduction of the moisture evaporation temperature may be caused by the conformation change of SF from random coil to *β*-sheet structure that enhanced the intermolecular hydrogen bonds between SF and CS molecules and reduced the interaction between SF and water. The downward shifting of the moisture evaporation which reaches maximum with the CF 1 : 2 blend film corresponded with the FTIR analysis that structural conformation was markedly displayed in this blend proportion.

The scaffold should have sufficient mechanical properties to maintain its structure during cell growth and implantation process by its nature of SF that normally has high mechanical strength. However, brittleness of a pure SF film is the limitation, and blending with CS is an alternative to improve the property of fibroin film. SF contains large proportion of hydrophobic domain with short-chain amino acids (Ala and Gly) which are packed and arranged to form *β*-sheet structure and resulted in its high strength [13, 38]. The result showed that prepared CS/SF blend films with higher proportion of fibroin resulted in higher brittleness (lower flexibility) that is difficult to withstand handling. Addition of CS may enhance flexibility of the blend films by interposition of CS along the fibroin chain which disturbs the close packing of the fibroin structure. However, the prepared CS/SF blend films showed higher strength and higher flexibility compared to the result stated previously [21]. This effect might be attributed to the solvent system dissolving CS and the difference in preparing regenerated SF. Lactic acid was used in this work to dissolve CS and SF. It was reported that lactic acid produced plasticizing effect improving the flexibility of the CS when compared to the general common solvent, acetic acid [29, 39, 40]. Furthermore, the regenerated SF in this work was prepared according to Miyaguchi's method by using calcium chloride solution and was suggested that the regenerated SF contained more *β*-sheet structure which might result in higher mechanical properties [26].

The water absorption ability reflects capability of the scaffold in holding aqueous medium which is necessary for the cell growth. The prepared CS/SF blend films could retain their shape after being immersed in an aqueous solution maintaining stable size and shape during cell culture or implantation process. By considering the possible applications of the blend films in skin tissue engineering, the prepared biomaterial exhibited appropriate swelling capacity because the absorption of fluid was approximately 80 times of its initial weight. This is considered high enough for skin tissue engineering [41]. The water absorption ability is affected by free hydrophilic groups (–COOH, –NH_2_ and –OH). Therefore, hydrogen bonding interaction between C=O and N-H in CS and amide group of fibroin chains could dominate their swelling capacity. Corresponding with the FTIR and DSC analysis, the blend film with high CS content exhibited the highest water absorption due to the lower intermolecular hydrogen bonding between CS and SF molecules, which increased the interaction with water molecules.

Biodegradation of biomaterials is a promising function of a carrier. However, materials with rapid degradation are not suitable for application in tissue engineering. Our results showed higher degradation rate in films of high CS content (CF 3 : 1). This might be due to the high ability of water absorption in CS, which facilitates penetration of lysozyme to react with CS. Regarding the enzymatic degradation of CS, there is inverse relationship between degradation rate and deacetylation degree (DD) [42, 43]. Because of the coexistence of both amorphous and crystalline zone in CS molecule, CS with lower DD contains more acetyl groups and might have more amorphous region which promotes lysozyme penetration leading to faster degradation [43]. In this study, 95% deacetylated CS was used and therefore all of the blend films had weight remaining over 90%. This is comparable to results reported by Nwe et al. [27]. Prolongation of the scaffold can facilitate implantation in patients and replacement by neotissue from patients [44]. Our results indicated the possibility of using the CS/SF blend films as scaffold for the construction of skin equivalent by culturing at least 4 weeks to ensure that the films would not be completely degraded during the construction process.

The capability of the CS/SF blend films to support viability and proliferation of the fibroblast cells as a function of time is demonstrative of the cytocompatibility and feasibility for tissue engineering application. The results obtained from *in vitro *cytotoxicity study showed that the CS/SF blend films did not contain products toxic to cells and proliferation of the fibroblast cells on the blend film increased regularly with increasing cultivation time. The SEM image revealed spindlelike shape of the attached cells with filopodia-like extension adhering with the scaffold and connecting to adjacent cells. The evidence of cell-to-cell interaction and cell spreading can be considered as signs of healthy cells and indicative for noncytotoxic response of the cells on supporting material [27, 45]. The adhesion and proliferation of the cells on blend films may involve the interaction between negative groups on the cell surface and the remaining positive amino group on the blend scaffold [27, 46]. These results go together with the proliferative processes of cells on a matrix that begin with cells that adhere on the matrix, then spread and finally proliferate and differentiate [47]. Fibroblast cells are cell population in dermis responsible for production of new extracellular matrix, mainly consisting of collagen, to provide the strength to the repaired skin [48]. In dermis, about 80% of collagen are type I [49]. Beside cell adhesion and proliferation, fibroblast cells cultured on all the CS/SF blend films are active and could maintain their important functions by expressing collagen type I gene. These data indicated that the prepared blend scaffold can be used for construction of dermal substitute to support epidermal growth in the prepared skin equivalent.

 In summary, the CS/SF blend films were prepared and the FTIR and DSC analysis showed intermolecular interaction between CS and SF. The mechanical properties, swelling property, and degradation of the CS/SF blend films were affected by the proportion of CS and SF. These CS/SF blend films had no cytotoxicity and could support the growth of fibroblast cells as well as maintain cell functions. The cytocompatibility and appropriate physicochemical properties of CS/SF blend films indicated promising use in further study for skin tissue engineering application, for instance, using as supporting materials for construction of epidermodermal skin substitute.
